# Effectiveness of education and attitudes toward different types of deceased donor kidneys: Survey analysis of single-center experience

**DOI:** 10.3389/fpubh.2023.1116823

**Published:** 2023-03-29

**Authors:** Sumi S. Nair, Andrea N. Thorp, Wael Hanna, Bradley K. Johnson, Byron Smith, Savitha Iyengar, Elizabeth A. Howe, Girish K. Mour

**Affiliations:** ^1^Division of Nephrology, Mayo Clinic, Phoenix, AZ, United States; ^2^Department of Nursing, Mayo Clinic, Phoenix, AZ, United States; ^3^Dallas Nephrology Associates, Dallas, TX, United States; ^4^Division of Clinical Trials and Biostatistics, Mayo Clinic, Rochester, MN, United States; ^5^Manager Transplant Quality and Compliance, Mayo Clinic, Rochester, MN, United States

**Keywords:** education, outcomes, kidney, transplant, deceased donation, attitude, knowledge, public health

## Abstract

**Background:**

We lack data on the effectiveness of education and the patient's attitude toward different deceased donor kidney types. A prospective study was performed to evaluate patient attitudes, baseline knowledge, and effectiveness of our kidney transplant education process. We also analyzed the knowledge retention of our waitlist patients.

**Design:**

We prospectively surveyed a patient cohort using a paired analysis pre and post education with initial evaluation visit. Knowledge retention among waitlist patients was assessed with annual waitlist visit.

**Results:**

One hundred four patients received paired surveys to assess the baseline knowledge and effectiveness of education. Forty-three patients received a single survey with their annual waitlist evaluation to assess knowledge retention. Paired survey showed mixed results, with no statistically significant improvement in the kidney donor profile index domain. Significant improvement was seen in the hepatitis C virus–positive donor domain and the Public Health Service (PHS) increased-risk donor domain. For the waitlist cohort, overall knowledge retention ranged from excellent to fair, with a decline in knowledge for the PHS increased-risk donor domain.

**Conclusion:**

Our study suggests that the education intervention regarding different deceased donor kidney types is effective overall and transplant candidates retain the knowledge while waiting for transplant.

## Introduction

Organ shortages and longer wait times for kidney transplant have prompted a wider use of deceased donor types. Acceptance of the deceased donor organ pool has undergone a substantial change in the past two decades with the advent of extended criteria for donor kidneys in the early 2000s to taking hepatitis C virus (HCV)-positive kidney donors in the current era. Kidney Donor Profile Index (KDPI) estimates the duration of post-transplant kidney function such that lower KDPI values is expected to have a longer post-transplant kidney survival (1). KDPI was introduced in 2014 as part of the new kidney allocation system to improve donor recipient matching. KDPI is given on a cumulative percentage scale. Recipients who are typically younger and are expected to have the highest expected post-transplant survival can avail lower KDPI donors that last the longest. Similarly, older recipients and recipients with shorter expected post-transplant survival can choose to list for high KDPI kidney (>85%) to decrease wait time to transplant for a better quality of life and survival benefit as compared to being on dialysis. The utilization rates of kidneys from a Public Health Service (PHS) increased-risk donor and HCV positive donor are also an issue resulting in high discard rates (2–6), despite successful transplant outcomes (7, 8), resulting in high graft life loss in years (9). However, the use of KDPI > 85% (concerns for shorter kidney transplant lifespan), the Public Health Service (PHS) increased-risk donor and HCV-positive donors (both concerning for infection transmission) remain a concern (10). Some of the differences are attributed to transplant center practices and patient preferences toward acceptance of the different deceased donor types.

Changes in the deceased donor organ pool have made it more complex for patients to understand what type of kidney they are being offered. For example, donors with a KDPI of 50% may also meet criteria for PHS increased risk and as HCV positive kidneys based on identified risk factors and laboratory results of the donor. To complicate matters, some transplant centers also accept acute kidney injury (AKI) and hepatitis B core antibody (HBcAb)-positive kidneys. So, a candidate could receive an organ offer for a kidney from a PHS increased risk donor that has a KDPI of 50%, AKI, HBcAb positivity, and HCV positivity. Even for a highly educated patient, this organ offer can be challenging to understand. The patient may turn down the organ offer due to perceived risks of the specific donor type rather than focusing on the benefits from organ transplant. The perceived risks could be related to a lack of insight regarding deceased donor kidney types.

The kidney transplantation process starts with a referral to transplant center. The referred patient is then evaluated by the transplant center to determine their transplant eligibility. As part of transplant evaluation, each transplant center provides transplant education, but the patient education process differs across centers. Baseline knowledge and attitudes toward different deceased donor kidneys among transplant candidates and the effectiveness of transplant education about the different donor types is lacking. Once a patient is approved for kidney transplant, then they are placed on waitlist till they get a matched donor for kidney transplant. The waitlist period can be in years prior to receiving a kidney transplant. During the time of waiting, a patient's medical condition may change which can affect their transplant eligibility. Each transplant center manages their waitlisted patients in a variable way which may include visit with transplant center at routine time intervals till transplant, have a telephone review with patient, or just chart review. Patient may or may not be given a reeducation regarding different deceased donor types during this visit.

Our hypothesis was that patients evaluated for transplant have limited knowledge of the different deceased donor types at baseline and the education provided by our transplant center is effective in improving their knowledge. Our secondary hypothesis was that the retention of the education provided during the transplant evaluation declines by the time the patient is seen for their annual waitlist visit.

The goals of the study were to assess the baseline knowledge of the deceased donor types when transplant candidates start the transplant evaluation, assess the effectiveness of our transplant education process, and to include a waitlist cohort to assess retention of knowledge.

## Materials and methods

We conducted a prospective survey study at the Mayo Clinic Transplant Center in Arizona as part of a management quality project from November 21, 2019, through February 10, 2020. This survey was IRB exempt as it was part of quality improvement project. Candidates being evaluated for solitary kidney transplant were included while candidates for multiorgan transplants were excluded. We also excluded candidates who failed to answer any question in the survey or did not complete the paired survey. Transplant candidates were 18 years of age or older and spoke and read English language. Questionnaires to assess knowledge about kidney transplantation vs. dialysis exist, however, we were not able to find any questionnaire that assesses patients' knowledge of different deceased donor types. Hence, we developed a survey using the Delphi method (the authors SSN, ANT, SI, EAH, and GKM took part in developing this survey questionnaire). Initially, AKI and HbcAb were included but then excluded in second round of Delphi process due to limited acceptance of these donors across the country. Once the authors agreed upon the final set of questions, the survey was presented during our monthly transplant center quality meeting and approved by the multidisciplinary transplant team to administer to patients. Pretesting was not performed on any patients prior to implementation of survey due to limited questions being surveyed. The entire eight-question self-administered survey (Box 1) was completed during the clinic visit and graded on whether thr participant answered each question correctly. We collected pretransplant data related to their end-stage renal disease, demographic characteristics, education level, depression score with the Public Health Questionnaire-9 (PHQ-9), anxiety score with Generalized Anxiety Disorder-7 (GAD-7), psychological assessment with Stanford Integrated Psychosocial Assessment for Transplant (SIPAT) score, insurance status, and Karnofsky Performance Status Scale score. GAD 7 is 21 point scoring system for generalized anxiety disorder. Each five point increase in score is associated with increasing anxiety requiring intervention. PHQ-9 is a 27 point scoring system for identying and diagnosing depression. Higher PHQ-9 score is associated with increasing depression severity. SIPAT score assesses psychological outcome post transplantion based on pretransplant factors and given to potential candidate who comes for transplant evaluation. A score of of <6 translates to an excellent candidate, 7–20 score is a good candidate, >20 is a minimal acceptable candidate, while a score of >39 is poor to high risk candidate.

Box 1 **Questionnaire of kidney transplant candidates.**

1. What does *a high-KDPI (>85%) kidney organ* mean to you?
  a. KDPI means that the kidney organ comes from a donor with increased chances of having hepatitis C, hepatitis B, or HIV
  b. KDPI are kidney organs that are of bad quality
  c. If I accept a high-KDPI kidney organ, I will likely be transplanted sooner, but the kidney may not last as long as a low-KDPI (≤85%) kidney
  d. I don't know

2. How many years, on average, does a high-KDPI (>85%) kidney organ last?
  a. 1–2 y
  b. 2–3 y
  c. 5–6 y
  d. 8 y or more

3. Are you aware of a hepatitis C option for kidney organs?
  a. Yes
  b. No

4. If you get a hepatitis C–positive kidney, are you aware that we can almost completely cure hepatitis C post-transplantation?
  a. Yes
  b. No

5. What is a Public Health Service (PHS) increased-risk donor?
  a. A donor who had diabetes
  b. A donor who was a smoker
  c. A donor who had a behavior that increased his/her chances of having hepatitis C, hepatitis B, or HIV

6. Kidney organs from PHS increased-risk donors will not last as long or be as healthy
  a. True
  b. False

7. Would you accept a PHS increased-risk kidney organ if offered?
  a. Yes
  b. No
  c. Undecided

8. What is the risk of getting hepatitis B, hepatitis C, or HIV from a PHS increased-risk kidney organ?
  a. Low (<1%)
  b. Moderate (1–5%)
  c. High (>5%)

KDPI, kidney donor profile index.

As a part of the routine transplant evaluation at our center, we conduct two clinic visits with a transplant nephrologist. The first visit (*evaluation visit*) reviews the patient's health history, any prior transplants related results, and any initial patient concerns or questions regarding the evaluation or transplantation. The second visit (*wrap visit*) involves a review of all transplant testing completed during their evaluation and addresses any remaining concerns. All patients had their evaluation and wrap visits during this study period. Surveys were given to transplant candidates before both clinic visits.The evaluation visit was used to assess baseline knowledge. The candidate then received education provided by the transplant clinician during the initial clinic evaluation visit, along with a formal face to face education class as part of the transplant evaluation. The class was a 1-h based group session conducted by a transplant coordinator. The survey was given again before the wrap visit to assess the effectiveness of the education. This occurrence was our paired visit assessment (*evaluation/wrap visit*).

Our center routinely conducts annual clinic visit for our waitlist patients (*waitlist visit*) based on our center criteria. The waitlist patients had already undergone evaluation in the past and were awaiting for kidney transplant. The waitlisted patients were different as compared to our paired patients. A survey was given to these patients to assess their retention of knowledge regarding different deceased donor types from the initial evaluation. This was a 1-time survey that was conducted either prior to the waitlist clinic visit or the waitlist education class.

For all the above visits, a PowerPoint presentation is used for education. Handouts of the slides and education materials were provided for each candidate. Education includes review of the transplant evaluation process, benefits /risks of transplantation, medication regimen and side effects and the different donor types. The information about the different deceased donor types that is provided during our all of the transplant education classes has remained stable during this survey. The patients were identified based on the unique clinic visit type associated with each visit.

### Statistical analysis

Surveys were graded on whether the participant answered each of the eight questions correctly, yielding a score ranging from 0 to 8. Participants who answered no survey questions were excluded. Those who left some questions unanswered were included, but the unanswered questions were not entered in the analysis. A χ^2^ test was used to compare the overall correctness between time points of evaluation, wrap, and waitlist visits. An analysis of covariance was conducted on each question to determine the effect of predictor variables on the correctness of the questions' answers at the wrap visit, removing the effect of the correctness at evaluation. Univariate models were fit for all variables of interest, as well as multivariate models containing all variables of interest. A *post-hoc* power analysis was also done to determine that our models did have enough statistical power.

## Results

**Figure 1:**
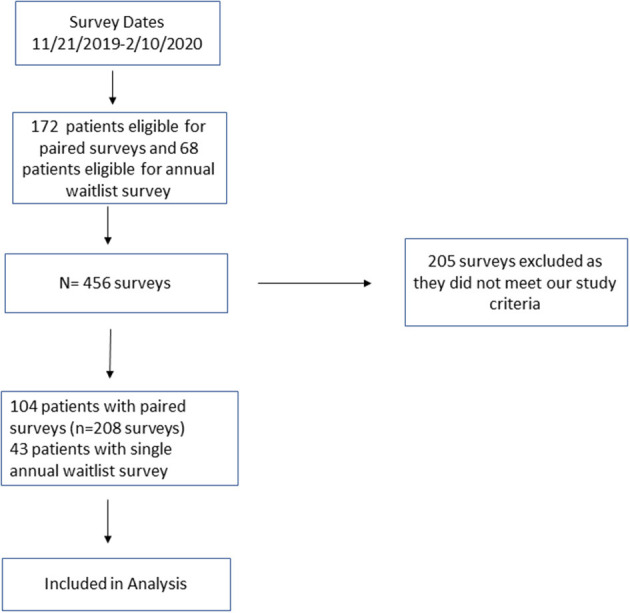
Flowchart of participant enrollment.

One hundred four patients responded to paired surveys (208 surveys) to assess baseline knowledge and effectiveness of education. Forty-three patients responded to a single survey with their annual waitlist visit (Figure 1).

Baseline demographic characteristics are outlined in Table 1 for paired patients and waitlist patients. Body mass index was significantly higher in waitlist patients (*P* < 0.006) than paired patients. Dialysis vintage was significantly greater in waitlist patients (mean, 1,980.5 days vs. 1,200.8 days; *P* = 0.01).

**Table 1:** Baseline demographic characteristics between paired and waitlist patient groups.

	**Groups** ^**a, b**^			
**Characteristic**	**Paired (eval/wrap) (*****n*** = **104)**	**Waitlist (*****n*** = **43)**	**Total (*****N*** = **147)**	***P*** **-value**
Age, mean (SD), y	56.1 (14.0)	57.7 (11.6)	56.6 (13.4)	0.53
Female sex	43 (41.3)	13 (30.2)	56 (38.1)	0.21
Race/ethnicity				0.26
African American	12 (11.5)	3 (7.0)	15 (10.2)	
Asian	12 (11.5)	2 (4.7)	14 (9.5)	
Hispanic/Latino	30 (28.8)	9 (20.9)	39 (26.5)	
Native American	3 (2.9)	1 (2.3)	4 (2.7)	
White	47 (45.2)	28 (65.1)	75 (51.0)	
BMI, mean (SD)	28.6 (5.2)	31.4 (6.3)	29.4 (5.7)	0.006
Dialysis vintage, mean (SD), d	1,200.8 (1,164.2)	1,980.5 (1,823.0)	1,408.7 (1,403.2)	0.02
Dialysis type				0.05
Hemodialysis	40 (63.5)	19 (82.6)	59 (68.6)	
Peritoneal	21 (33.3)	2 (8.7)	23 (26.7)	
Previous transplant organ				0.30
Kidney	7 (100.0)	6 (85.7)	13 (92.9)	
Liver	0 (0.0)	1 (14.3)	1 (7.1)	
ESRD				0.73
Diabetes mellitus	41 (39.4)	15 (34.9)	56 (38.1)	
Glomerular	24 (23.1)	8 (18.6)	32 (21.8)	
Hypertension	14 (13.5)	6 (14.0)	20 (13.6)	
Other	25 (24.0)	14 (32.6)	39 (26.5)	
State of residence				0.004^c^
Arizona	51 (49.0)	34 (79.1)	85 (57.8)	
California	29 (27.9)	5 (11.6)	34 (23.1)	
Other	24 (23.1)	4 (9.3)	28 (19.0)	
PHQ-9 score, mean (SD)	3.7 (4.1)	3.5 (4.1)	3.7 (4.1)	0.73
GAD-7 score, mean (SD)	2.7 (4.1)	2.5 (3.5)	2.6 (3.9)	0.81
SIPAT group				0.37
1 (SIPAT ≤6)	13 (12.9)	8 (18.6)	21 (14.6)	
2 (6 <SIPAT ≤20)	58 (57.4)	27 (62.8)	85 (59.0)	
3 (20 <SIPAT ≤39)	26 (25.7)	8 (18.6)	34 (23.6)	
4 (SIPAT > 39)	4 (4.0)	0 (0.0)	4 (2.8)	
Education level				0.54
Elementary school	4 (3.8)	1 (2.3)	5 (3.4)	
High school	48 (46.2)	14 (32.6)	62 (42.2)	
College	39 (37.5)	21 (48.8)	60 (40.8)	
Post-college	9 (8.7)	4 (9.3)	13 (8.8)	
Other	4 (3.8)	3 (7.0)	7 (4.8)	
Karnofsky score				0.54
≤70	55 (54.5)	21 (48.8)	76 (52.8)	
>70	46 (45.5)	22 (51.2)	68 (47.2)	
Work status				0.62
Employed	40 (41.7)	16 (37.2)	56 (40.3)	
Unemployed	56 (58.3)	27 (62.8)	83 (59.7)	
Insurance				0.80
Private	41 (39.4)	16 (37.2)	57 (38.8)	
Public	63 (60.6)	27 (62.8)	90 (61.2)	
Listed at another transplant center	22 (21.2)	2 (4.7)	24 (16.3)	0.01

BMI, body mass index; ESRD, end-stage renal disease; eval, evaluation; GAD-7, Generalized Anxiety Disorder-7; Karnofsky, Karnofsky Performance Status Scale; PHQ-9, Patient Health Questionnaire-9; SIPAT, Stanford Integrated Psychosocial Assessment for Transplant.

^a^Unanswered survey questions were not entered in the analysis.

^b^Values are presented as number (percentage) of patients unless specified otherwise.

^c^A difference exists between the proportion of the state's people who lived in between the groups (wrap visit vs. waitlist visit). The difference is between the groups in Arizona and California. Arizona is represented more across both groups. California is different between the 2 groups (*P* = 0.03), and Arizona is different between the 2 groups (*P* < 0.001), whereas Other is not (*P* = 0.07).

A statistically significant number of paired survey patients were listed at another transplant center, perhaps because of an out-of-state residence and a desire to be listed at another center. The proportion of surveys from Arizona waitlist candidates was greater than the proportion in the paired analysis.

### Pre-education survey for initial evaluation

#### KDPI

1. What does *a high-KDPI (*>*85%) kidney organ* mean to you?
2. How many years, on average, does a high-KDPI (>85%) kidney organ last?

Sixty-eight percentage of patients knew that they may receive a transplant sooner if they were willing to accept a high-KDPI kidney. Only 58% knew about the average allograft survival of high-KDPI kidney organs.

#### Hepatitis C

3. Are you aware of a hepatitis C option for kidney organs?
4. If you get a hepatitis C–positive kidney, are you aware that we can almost completely cure hepatitis C post-transplantation?

Fifty-one percentage of patients were aware of the option to accept an organ from an HCV-positive donor, but 57% were aware of a cure for HCV.

#### PHS increased-risk donor

5. What is a Public Health Service (PHS) increased-risk donor?
6. Kidney organs from PHS increased-risk donors will not last as long or be as healthy.
7. Would you accept a PHS increased-risk kidney organ if offered?
8. What is the risk of getting hepatitis B, hepatitis C, or HIV from a PHS increased-risk kidney organ?

Questions five through eight assessed the patient's knowledge and attitude toward PHS increased-risk donors. Candidates had excellent knowledge about infection transmission risk, but they scored poorly regarding the infection transmission risk rates, and allograft survival. Less than 50% agreed to accept a PHS increased-risk donor organ.

### Pre- and post-education paired analysis for initial evaluation

**Figure 2:**
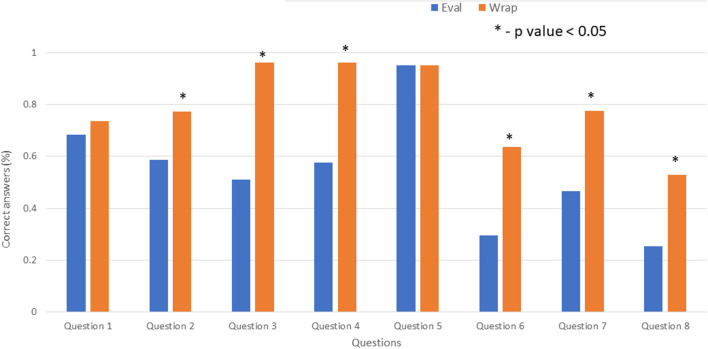
Comparison of evaluation (Eval) and wrap visits. Asterisk indicates *P* < 0.05. Eval, evaluation.

The median time between the evaluation and the wrap visit was 6 days (IQR-10.25 days). We saw an improvement from the pre-education assessment to the post-education assessment. The degree of improvement was statistically significant across all questions except questions 1 and 5 (Figure 2). The rate of correct response for question 5 was high before education, and it stayed stable post education. For question 1, some improvement was observed, but it was not statistically significant.

### Waitlist data

A high retention of knowledge regarding the different deceased donor types was observed, except for questions 6 through 8. The correct response rate for questions 6 and 8 and the willingness to accept PHS increased-risk organs (question 7) was 50% or lower, which was better than the initial baseline knowledge (evaluation visit) but worse compared with the post education intervention (wrap visit).

### Baseline knowledge (evaluation visit) vs. education intervention (wrap visit) vs. waitlist visit

**Figure 3:**
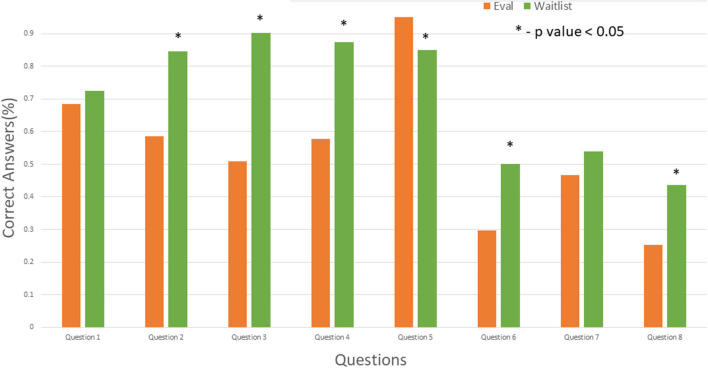
Comparison of evaluation (Eval) and waitlist visits. Asterisk indicates *P* < 0.05. Eval, evaluation.

**Figure 4:**
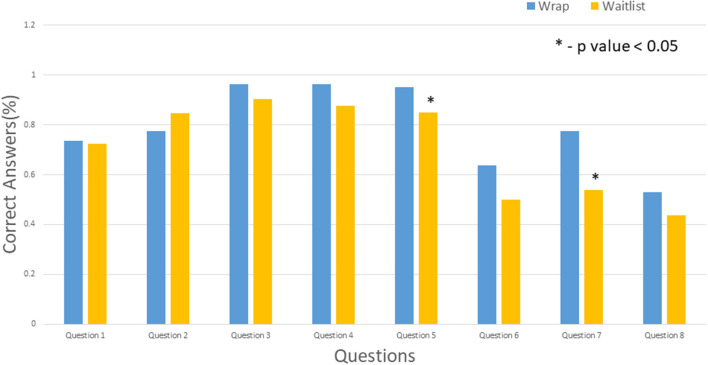
Comparison of wrap and waitlist visits. Asterisk indicates *P* < 0.05.

In comparison between the initial evaluation visit and waitlist patients, the results differed across the various questions, from statistically significant improvement in knowledge (questions 2, 3, 4, 6, and 8) to significant decline in knowledge (question 5) to no difference (questions 1 and 7) (Figure 3). However, comparison of the wrap visit with the waitlist visit showed that most candidates did not have a statistically significant difference except for significant decline in the answers to questions 5 and 7 (Figure 4).

### Predictors for education effectiveness in paired samples on evaluation/wrap visits

We used candidate-related factors to analyze the effectiveness of our education at the wrap visit in univariate and multivariate (Supplementary Table 1) models. Higher scores for depression on PHQ-9 (*P* = 0.004) with a cutoff value of 5 or above and for anxiety on GAD-7 (*P* = 0.01) with a cutoff value of 7 or above was significantly associated with decreased effectiveness of education for question 1. Residency in “other” states was associated with higher scores at the wrap visit for question 6. This difference in scores could be due to prior education from a different transplant center and the education provided during our initial evaluation was essentially a refresher course for these candidates. College education (*P* = 0.04) was associated with increased acceptability of PHS increased-risk donors for question 7. Public insurance was negatively associated (*P* = 0.02) and a higher Karnofsky score was positively associated (*P* = 0.008) with the effectiveness of education for question 8.

However, in the multivariate analysis, no specific variable predicted the effectiveness of education except for question 7. College education (*P* = 0.04) was associated with increased acceptability of PHS increased-risk donors (Supplementary Table 1).

### Patient outcomes for donor listing based on evaluation/wrap visits

Sixty-eight patients were listed for kidney transplant. For the remaining 36 patients, a transplant was either denied or the patients were still in the evaluation phase at the time of manuscript writing. We do not have data on PHS increased-risk donor listing status because our center's custom is to consent for the PHS increased-risk option at the organ offer instead of at the time of listing. Among the listed candidates, 29 were listed as willing to accept high-KDPI kidney organs and 39 declined such kidneys, which is consistent with the United Network for Organ Sharing (UNOS) Benchmark Report. In addition, 50 candidates were listed as willing to accept HCV positive kidneys, and 18 patients declined HCV positive kidney donor. We did not analyze donor Hepatitis C listing for the waitlist candidates because they were already listed prior to initiation of study.

## Discussion

The outcomes of the education intervention were impressive, with statistically significant improvement in knowledge in most of the assessed parameters. We went a step further to assess retention of knowledge for our waitlist candidates. Knowledge retention as assessed during their annual waitlist visit stayed high. The knowledge regarding PHS increased-risk donors continues to be an issue both with pre-education evaluation visit and with the annual waitlist visit.

The focus in most transplant studies has been the survival benefits of transplant (11–13). Studies are lacking on the transplant candidate's knowledge about different deceased donor types despite changes in designation of deceased donors over last two decades. Our survey is distinctive as it assesses the baseline knowledge and attitudes about different deceased donor types before formal education provided by our center. The survey further evaluated the effectiveness of the education provided by our center through a paired survey analysis. More importantly, we do not know whether the transplant candidates retain the knowledge about different deceased donor types during their waitlist period, a detail that can have a serious effect on decision-making because of long wait times for transplant across the country. Our results show high retention rates though with additional education, a transplant candidate's knowledge about donors who meet PHS risk criteria can be improved. This may require more frequent reminders instead of reliance on a yearly refresher. Additionally, we did analyze previously transplanted patients and found no difference in paired analysis or as compared to waitlist patients (data not shown).

We did not know whether patients had received any education before their transplant evaluation. However, educators at dialysis units are ineffective in providing transplant related education, discuss different donor types or provide education materials (14). In our study, their baseline knowledge was at an acceptable level. We found a 68% improvement in HCV treatment knowledge and a 66% increase in willingness to accept PHS increased-risk organs after education. Improvement was favorable across all domains, with statistical significance for most domains.

Racial and socioeconomic differences can affect a candidate's ability to complete the transplant evaluation but may be modified by education (15). In our study, socioeconomic status did not make a difference in the education intervention besides the willingness of college-educated candidates to accept PHS increased-risk donors. However, higher PHQ-9 and GAD-7 scores was associated with reduced effectiveness of education for the high-KDPI domain. The latter implies that even mild degrees of depression and anxiety can decrease the effectiveness of the education and can be addressed with intervention.

The discard rates of kidneys from a PHS increased-risk donor remains an issue (2–4). Education interventions may not translate into willingness to accept PHS increased-risk kidney organs, and knowledge retention may decrease later (16). Ninety-five percentage of the candidates understood the definition of PHS increased risk pre education. However, wide variability was seen in the understanding of risk of infectious disease transmission, the quality and longevity of the donated organ, and the willingness to accept this organ type. Our study showed that with education, significant improvement in knowledge and willingness to regarding PHS increased-risk donors can be achieved. Unfortunately, this did not translate into retention as shown by a decline during the waitlist period. This outcome alludes to lingering concerns for the use of PHS increased-risk donor kidneys despite having increased long-term survival benefit and possibly getting a kidney transplant with lower KDPI (17). This could be also due to allied health staff's discomfort regarding PHS increased risk donors due to possible lack of knowledge for the increased-risk donor, based on behavior (18). The willingness to accept PHS increased donors can be further enhanced with involvement of community nephrologist (19) besides the transplant physician. Given concerns for the potential underuse of PHS increased-risk organs, UNOS policy was changed on March 1, 2021 (20).

In our study, >50% of pre-education patients and 90% of waitlist patients were aware of the HCV-positive donor option. This was a surprising finding in our waitlist cohort because our transplant program had started accepting HCV-positive donors only a few months before implementing the survey. It means that either other resources were available for our waitlist patients or acquiescence bias occurred because it was simply a yes/no question. The finding also showed the greatest improvement after education, which could be secondary to the emphasis on shorter waiting time for HCV-positive donors. In addition, it showed patient attitudes toward accepting HCV-positive donors, as opposed to accepting PHS increased-risk donors. The candidates may feel comfortable in knowing about a curative treatment for HCV.

### Limitations

Our study is limited due to single center experience and small sample size. The survey was created by study team since none exist in literature and not validated. We realize underlying health literacy may play a part in patients' ability to understand health care. However, our focus was on our effectiveness of our education process irrespective of underlying health literacy and did not assess the same. Our study included English-speaking patients only. We did not analyze various racial subgroups because of our small sample size. We did not explore the reasons behind the lack of improvement among patients who continued to have limited understanding about the different donor types.

### Conclusion

Our study focused on the knowledge and attitudes toward different deceased donor types and found acceptable baseline knowledge except for PHS increased-risk donor types. We also determined that the knowledge improved significantly after the education intervention, with excellent retention during the waitlist period. Despite above limitations, the education we provide to our patients continue to be our strongest tool for improving candidate awareness about transplant, including the types of deceased donor kidneys. We suggest frequent reinforcement of transplant education during the waitlist period for further improvement in and retention of knowledge regarding deceased donor types.

## Data availability statement

The original contributions presented in the study are included in the article/Supplementary material, further inquiries can be directed to the corresponding author.

## Ethics statement

Ethical review and approval was not required for the study on human participants in accordance with the local legislation and institutional requirements. Written informed consent from the participants was not required to participate in this study in accordance with the national legislation and the institutional requirements.

## Author contributions

SN: project conception, questionnaire development, data collection and analysis, and manuscript writing and editing. AT and EH: data collection and analysis and manuscript writing. WH: questionnaire development, data collection, and manuscript writing. BJ and BS: project statistician, data analysis, and manuscript writing. SI: data analysis and manuscript writing. GM: project conception and questionnaire development, data collection and analysis, manuscript writing and editing, and overall supervisor for the project. All authors contributed to the article and approved the submitted version.
