# Supplementary material for: Effectiveness of education and attitudes toward different types of deceased donor kidneys: Survey analysis of single-center experience
Source: Front Public Health. 2023 Mar 29;11:1116823. doi: 10.3389/fpubh.2023.1116823 (PMC10090271; doi:10.3389/fpubh.2023.1116823)
Supplement: Supplementary file 1 [file Data_Sheet_1.PDF]

Supplementary Table 1: Models

Univariate Model Analysis of Selected Characteristics				
	**Question No., Coefficient (SE)**			
Characteristic	1	6	7	8
State- other than AZ		1.2[a] (0.57)		
PHQ-9 score	-0.2[a] (0.07)			
GAD-7 score	-0.2[a] (0.06)			
College Education			2.8[a] (1.32)	
Karnofsky score >70				1.2[a] (0.44)
Insurance, public				-1.0[a] (0.43)
Multivariate Analysis Model of Selected Characteristics				
College Education			3.3[a] (1.63)	

Supplementary Table 1.

Abbreviations: GAD-7, Generalized Anxiety Disorder-7; PHQ-9, Patient Health Questionnaire-9, Karnofsky, Karnofsky Performance Status Scale.

Reference variables for each grouping are state, Arizona (AZ) (compares CA vs AZ and Other vs AZ); education level, elementary; Karnofsky score, ≤70; work status, employed; insurance, private.

[a] Statistically significant value with $p < 0.05$
